# The Education of the Consumptive

**Published:** 1903-11-21

**Authors:** 


### The Education of the Consumptive.

Between the optimism of the few and the pessimism of the many there is usually a place for the hopeful worker. Pulmonary tuberculosis is now known to be not only a preventable disease but one capable in many subjects, provided suitable conditions be available, of undergoing such arrest as to be considered, for practical purposes, curable. But for the great bulk of consumptive sufferers adequate treatment is unattainable, and as far as we can see it will be long before anything like a sufficient number of suitably equipped sanatoria, where rational hygienic treatment can be systematically carried out, will be available for poor cases in this country. It becomes therefore a matter for the most serious consideration whether a more sharply defined and vigorously conducted educational policy might not best meet the urgent necessities of the present situation. After a thorough study of the subject in all its aspects we are strongly of opinion that sufficient consideration is not being given to the all-important matter of teaching the consumptive how best to help himself, and in so doing to assist the State in a successful combat with what is indeed the white man's burden.

The task of instructing the consumptive is one of much difficulty. Satisfactory results can only be attained by patient, persistent, individual effort. Medical officers of health have long sought by the issue of public notices to turn the wayward, pathological sinner and sufferer into the path of hygienic rectitude. The London County Council in its recently adopted by-laws is seeking to enforce an educational reform in regard to expectoration even at the point of the law. Much quiet work is also being accomplished by visitors and district nurses and other philanthropic labourers among the poor. Many of those sufferers who have benefited from residence in a sanatorium become veritable missionaries of hygiene and ardent advocates of the doctrine of a salvation through open-air. It is probable that much more might be done in many sanatoria in securing for the patients a simple but complete understanding of the principles on which the hygienic conduct of their future life must depend. We are acquainted with one sanatorium where the doctor lectures once a week to the patients, and we are informed that the results are eminently satisfactory. The educational advantages which may accrue even from a comparatively short stay in a well-directed sanatorium cannot be exaggerated, and efforts carried out at the public expense, such as those conducted by Dr. Arthur Newsholme at the Brighton Borough Sanatorium, are deserving of serious attention. Much also might be accomplished in the suitable training of consumptives by some such arrangement as that introduced by Dr. R. W. Philip at the Victoria Hospital for Consumption at Edinburgh, where "day boarders" are enabled to visit the institution and receive instruction in the conduct of the hygienic life. The opportunities for imparting instruction to the consumptives attending at dispensaries and visiting out-patient departments are oftentimes not made use of to the extent which is desirable in the interests of both patients and public.

The fact is we have been so taken up with the attractive work of establishing sanatoria for the consumptive few that we have to a great extent neglected the education of the consumptive many. The time is now near when serious effort must be made to educate all sorts and conditions of men and women, whether sufferers from consumption or not, in the hygienic principles on which must depend our best efforts to prevent as well as to arrest pulmonary tuberculosis.

